# Liver Venous Deprivation (LVD) Versus Portal Vein Embolization (PVE) Alone Prior to Extended Hepatectomy: A Matched Pair Analysis

**DOI:** 10.1007/s00270-022-03107-0

**Published:** 2022-03-21

**Authors:** Georg Böning, Uli Fehrenbach, Timo Alexander Auer, Konrad Neumann, Martin Jonczyk, Johann Pratschke, Wenzel Schöning, Moritz Schmelzle, Bernhard Gebauer

**Affiliations:** 1 Department of Radiology, Charité - Universitätsmedizin Berlin, corporate member of Freie Universität Berlin, Humboldt-Universität zu Berlin, and Berlin Institute of Health, Augustenburger Platz 1, 13353 Berlin, Germany; 2 Institute of Biometry and Clinical Epidemiology, Charité - Universitätsmedizin Berlin, corporate member of Freie Universität Berlin, Humboldt-Universität zu Berlin, and Berlin Institute of Health, Charitéplatz 1, 10117 Berlin, Germany; 3 Department of Surgery, Charité - Universitätsmedizin Berlin, corporate member of Freie Universität Berlin, Humboldt-Universität zu Berlin, and Berlin Institute of Health, Augustenburger Platz 1, 13353 Berlin, Germany; 4 Berlin Institute of Health (BIH), Anna-Louisa-Karsch-Straße 2, 10178 Berlin, Germany

**Keywords:** Liver venous deprivation (LVD), Portal vein embolization (PVE), Right hepatic vein embolization (rHVE), Future liver remnant (FLR), Extended hepatectomy

## Abstract

**Background:**

To investigate whether liver venous deprivation (LVD) as simultaneous, portal vein (PVE) and right hepatic vein embolization offers advantages in terms of hypertrophy induction before extended hepatectomy in non-cirrhotic liver.

**Materials and Methods:**

Between June 2018 and August 2019, 20 patients were recruited for a prospective, non-randomized study to investigate the efficacy of LVD. After screening of 134 patients treated using PVE alone from January 2015 to August 2019, 14 directly matched pairs regarding tumor entity (cholangiocarcinoma, CC and colorectal carcinoma, CRC) and hypertrophy time (defined as time from embolization to follow-up imaging) were identified. In both treatment groups, the same experienced reader (> 5 years experience) performed imaging-based measurement of the volumes of liver segments of the future liver remnant (FLR) prior to embolization and after the standard clinical hypertrophy interval (~ 30 days), before surgery. Percentage growth of segments was calculated and compared.

**Results:**

After matched follow-up periods (mean of 30.5 days), there were no statistically significant differences in relative hypertrophy of FLRs. Mean ± standard deviation relative hypertrophy rates for LVD/PVE were 59 ± 29.6%/54.1 ± 27.6% (*p* = 0.637) for segments II + III and 48.2 ± 22.2%/44.9 ± 28.9% (*p* = 0.719) for segments II–IV, respectively.

**Conclusions:**

LVD had no significant advantages over the standard method (PVE alone) in terms of hypertrophy induction of the FLR before extended hepatectomy in this study population.

## Introduction

Right (extended) hepatectomy is a complex surgical procedure for resecting hepatic metastasis in the right liver lobe and segment 4 or central tumors such as perihilar cholangiocarcinoma (CC). To ensure adequate liver function after surgery, various aspects must be taken into account including the future liver remnant (FLR) volume. To prepare patients before extended liver resection, two techniques to increase the FLR volume by induction of hypertrophy are established: portal vein embolization (PVE) and associating liver partition and portal vein ligation (ALPPS) of the projected resection volume [1]. In general, portal vein embolization (PVE) is performed a few weeks before hepatectomy [2, 3]. Various techniques and materials are available for PVE, but most investigators prefer a percutaneous transhepatic access using ultrasound-guided puncture and glue or particles [4]. Alternative access options are the intraoperative portal vein access, the transjugular intrahepatic portosystemic shunt (TIPS) like transjugular access and the transsplenic access to the portal vein [5]. Following the hypertrophy period, imaging is performed before surgery to confirm an adequate volume increase and to estimate the FLR.

Improving the hypertrophy could achieve a larger FLR volume within the same time interval and thus reduce the risk of postoperative liver function impairment. Alternatively, an improved hypertrophy could theoretically be used to shorten the interval to achieve the target FLR volume, thus reducing the risk of preoperative disease progression, especially in patients with aggressive tumors.

One possible approach to increase hypertrophy of the FLR is to perform right hepatic vein embolization (rHVE) in addition to PVE [6, 7]. HVE and PVE used to be done sequentially but nowadays are probably performed as simultaneous liver venous deprivation (LVD) in most centers. Transjugular or percutaneous/transhepatic access routes are possible [8]. In an animal model, no significant benefit was observed after a short interval of a few days [9]. On the other hand, promising results have been found in a mostly retrospective clinical analysis of sequential PVE and HVE [10].

The aim of this study therefore was to prospectively investigate the relative hypertrophy rate of the FLR after simultaneous LVD compared with the standard method of PVE alone.

## Materials and Methods

### Study Population

**Fig. 1:**
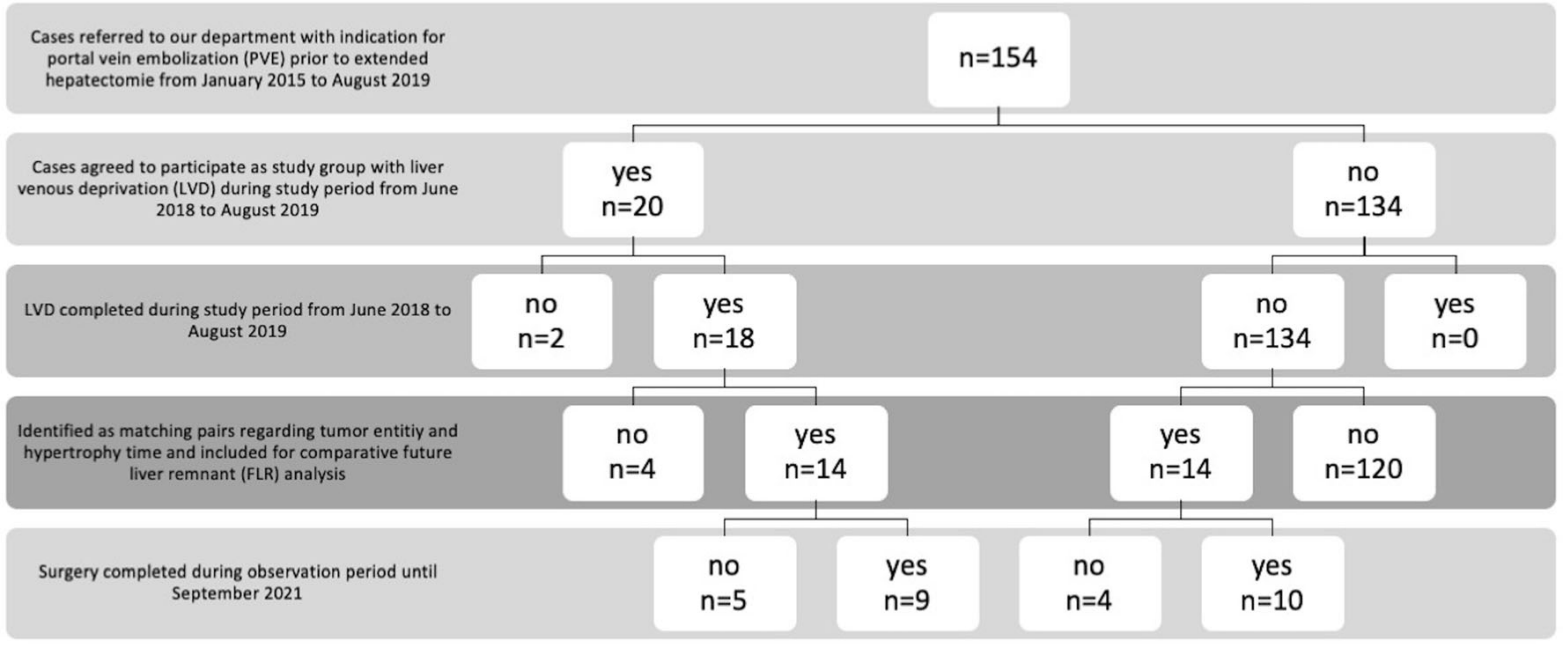
Flowchart study population. From June 2018 to August 2019, 20 patients with an indication for extended right hepatectomy and PVE at this center agreed to participate as study group with LVD. Two patients had to be excluded from LVD—one patient withdrew his consent to rHVE during the intervention and one patient could not be treated due to hepatic abscess formation related to a septic event, resulting in 18 completed LVDs. Subsequently, 134 consecutive patients who underwent PVE before extended right hepatectomy were retrospectively screened from January 2015 to August 2019 for direct matching regarding hypertrophy time (defined as time from embolization to follow-up imaging) and tumor entity. 14 matches were identified and loss of 4 LVD cases was accepted. There was no significant difference regarding liver resection rates between the groups (PVE: 10 of 14, LVD: 9 of 14, *p* = 0.500). In all other cases where surgery was not completed, tumor progression occurred during the hypertrophy interval, and systemic therapies were used instead. Portal vein embolization (PVE), liver vein deprivation (LVD), right hepatic vein embolization (rHVE), future liver remnant (FLR)

This prospective, single-center study was approved by the local ethics committee (EA2/073/18). From June 2018 to August 2019, 20 patients with an indication for extended right hepatectomy and PVE at this center agreed to participate as study group (Fig. 1). Written informed consent was obtained. Two patients had to be excluded from LVD—one patient withdrew his consent to rHVE during the intervention and one patient could not be treated due to hepatic abscess formation related to a septic event.

Subsequently, 134 consecutive patients who underwent PVE before extended right hepatectomy were retrospectively screened from January 2015 to August 2019. The matching algorithm described in the statistical analysis section identified 14 matches [11]. Since a further extension of the screening period for the control group would have identified patients undergoing PVE with use of a relevantly different interventional technique, loss of 4 LVD cases was accepted.

The following patient characteristics were recorded: sex, age, height, weight, tumor entity/pathological diagnosis, hypertrophy time (defined as interval from embolization to first control CT), whether surgery was completed, presurgical maximum liver function capacity (LiMAx) score (Humedics, Berlin, Germany) and whether the patient had postsurgical liver failure or died during the observation period until September 2021 including underlying causes [12–14]. To be accepted for embolization at this department, patients need to meet the following criteria: platelet count > 50,000/nl, partial thromboplastin time (PTT) < 50 s and prothrombin time (PT), resulting in an international normalized ratio (INR) < 1.5.

### Technique of LVD

Most interventions were performed under analgosedation. If patients preferred full sedation, general anesthesia was used in selected cases.

PVE was performed using the same technique in both groups following this institution’s standard of care using coils, particles and rarely plugs as described elsewhere [4]. The patients in this study were treated by four very experienced interventional radiologists with more than 10 years of experience inter alia in PVE. Because LVD was not performed as a standard procedure at this center before the start of the study, there was no relevant experience in this regard.

**Fig. 2:**
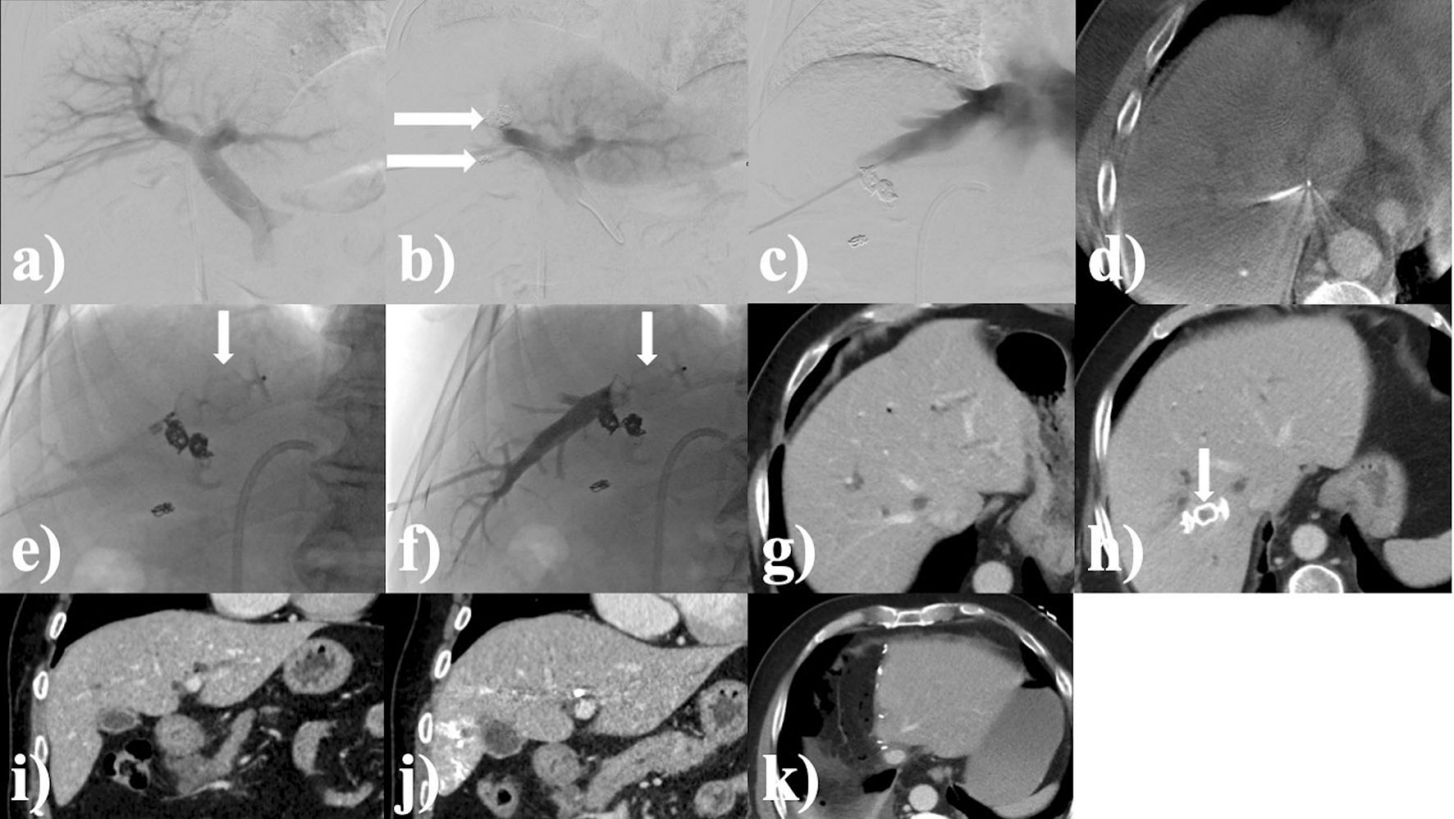
Example case. A 75-year-old man with cholangiocarcinoma (CC, Klatskin tumor type IIIa). Total radiation dose applied during intervention: 64.6 Gy*cm^2^, fluoroscopic time of intervention: 17 min; hypertrophy time: 28 days; relative hypertrophy rate of liver segments II + III: 68.2%, relative hypertrophy rate of liver segments II–IV: 68.1%; LiMAx score before surgery: 291 µg/h/kg. **a** Digital subtraction angiography (DSA) of the portal vein before embolization (PVE) after ultrasound-guided, transhepatic puncture. **b** DSA portography after embolization of the right portal vein branch with coils and particles. **c** DSA venography of the right hepatic vein after ultrasound-guided transhepatic puncture. **d** Verification of catheter position in the right hepatic vein by cone-beam CT. **e** Fluoroscopic positioning of the plug in the central right hepatic vein. **f** DSA of embolization of the puncture tract including peripheral vascular segments with a mixture (ratio of 2:1) of *n*-butyl-2-cyanoacrylate and ethiodized oil. **g** Planning CT before LVD in venous contrast phase. **h** CT in venous phase after hypertrophy time with verification of correct plug positioning in the right hepatic vein. **i** Planning CT before intervention in coronal orientation. **j** CT after hypertrophy time in coronal orientation with subjective hypertrophy of the left liver lobe. FLR hypertrophy (segments II + III) in this case was 68%. **k** CT after extended right hepatectomy horizontal arrows: coils, vertical arrows: plug

After successful PVE, a percutaneous, transhepatic approach was also chosen for simultaneous rHVE, if judged reasonably practicable by the interventional radiologist. Otherwise (e.g., tumor manifestation within the planned puncture tract), a transjugular approach was chosen and embolization performed using the individually preferred materials. We performed rHVE with a plug (AMPLATZER^™^ Vascular Plug II, St. Jude Medical, Saint Paul, USA). In a phlebography of the right hepatic vein, the diameter near the junction with the inferior caval vein was measured. A 30% oversized plug was deployed via an appropriate sized marked sheath near the junction with the inferior caval vein. After plug implantation, the puncture tract, including peripheral vascular segments of the right liver vein, was embolized with a mixture (2:1 ratio) of *n*-butyl-2-cyanoacrylate (Histoacryl^®^, B. Braun, Melsungen, Germany) and ethiodized oil (Lipiodol^®^ Ultra-Fluid, Guerbet, Bloomington, USA) (Fig. 2). When using a transjugular route, a 5F multipurpose (MP) catheter (TEMPO™, Cordis, Santa Clara, USA) was placed distally into the right liver vein via a large bore sheath before plug implantation for administration of the mixture to the right liver vein after plug positioning. The applied radiation dose was taken from dose reports. Complications were recorded according to the Cardiovascular and Interventional Radiological Society of Europe (CIRSE) guideline [15].

### Imaging and Volumetric Measurement

All patients underwent contrast-enhanced imaging (CT or MRI) before embolization and a contrast-enhanced CT scan after the standard clinical hypertrophy interval (~ 30 days) at this institution. The dates of imaging were recorded in each case. Imaging was performed with a maximum slice thickness of 5 mm. All volumetric measurements were performed manually by the same experienced reader (> 5 years gastrointestinal/abdominal imaging) using a commercially available software tool (Visage 7, Visage Imaging GmbH, Berlin, Germany). Large structures such as vessels were excluded. The reader measured the volumes of liver segments II, III, IV, II–IV, I + V–VIII and total liver volume. The measured absolute volumes were used to calculate relative hypertrophy rates for each case as follows: ((volume after hypertrophy interval-volume directly before intervention)/volume directly before intervention)*100.

### Statistical Analysis

Statistical analysis was performed using IBM SPSS Statistics 26 for Windows 10 (IBM Corp., Armonk, NY; USA) and R version 4.0.5. Matching was performed using the “Match” function from R’s “Matching” package (version 4.9–7) [11]. The criteria for direct matching were hypertrophy time (defined as time from embolization to follow-up imaging) and tumor entity. For hypertrophy time, a caliper of 0.06 was applied, for tumor entity the matching was exact. Descriptive evaluation of the data was done using means and standard deviations (SD) for metrical variables and frequencies for categorical or ordinal variables. Metric variables are displayed in box plots. For confirmatory data analysis, we used Student’s t-test for paired samples for metrical variables. For categorial variables Fisher´s exact test was used. Since the study is exploratory, no adjustment for multiple testing was performed. A *p* value < 0.05 was considered statistically significant.

## Results

### Patient Characteristics

Patient characteristics of the study population and control group are summarized in Table 1. There were no significant differences in mean hypertrophy time, age, height and weight between patients of the two groups. CC was the most common tumor entity in both groups, because resection of perihilar CC is a frequently performed treatment at this center. All analyzed cases had enough liver growth for the proposed extended hepatectomy. In 19 of 28 patients, liver resection could be performed as scheduled, there was no significant difference regarding to liver resection rates (PVE: 10 of 14, LVD: 9 of 14, *p* = 0.500, Table. 1, Fig. 1). In all other cases where surgery was not completed, tumor progression occurred during the hypertrophy interval, and systemic therapies were used instead (PVE: *n* = 4; LVD: *n* = 5). No liver failure was observed after surgery. Causes of deaths occurring during the observation period until September 2021 were unrelated to embolization in either group.

**Table 1:** Characteristics of matched study patients and matched controls

	Intervention		*P *value
	PVE (matched controls, *n* = 14)	LVD (matched study population, *n* = 14)	
Sex			
male	10 (71.4%)	8 (57.1%)	0.695
female	4 (28.6%)	6 (42.9%)	
Entity/pathological diagnosis			
CC	10 (71.4%)	10 (71.4%)	exact match
CRC	4 (28.6%)	4 (28.6%)	
Hypertrophy time [days]	30.57 ± 6.86	30.50 ± 7.17	match with caliper of 0.06
Complications of embolization			
no	14 (100%)	12 (85.7%)	0.481
yes	0 (0%)	2 (14.3%)	
Surgery completed			
no	4 (28.6%)	5 (35.7%)	0.500
yes	10 (71.4%)	9 (64.3%	
Death within observation period until Sep. 2021			
no	9 (64.3%)	11 (78.6%)	0.678
yes	5 (35.7%)	3 (21.4%)	
Liver failure postsurgery			
no	14 (100%)	14 (100%)	–
yes	0	0	
Age [years]	65.1 ± 11.4	68.1 ± 10.5	0.503
Height [cm]	176.9 ± 8.8	173.1 ± 9.2	0.262
Weight [kg]	81.6 ± 12.4	73.1 ± 15.1	0.113
BMI [kg/m^2^]	26.1 ± 4.2	24.1 ± 3.6	0.222

Study and control patients with indication for PVE prior to extended hepatectomy were matched for tumor entity and hypertrophy time. Tumor entities were cholangiocarcinoma (CC) and colorectal carcinoma (CRC). There were no significant differences between the two groups of patients. In all cases where surgery was not completed, tumor progression occurred during the hypertrophy interval, and systemic therapies were used instead.

**Fig. 3:**
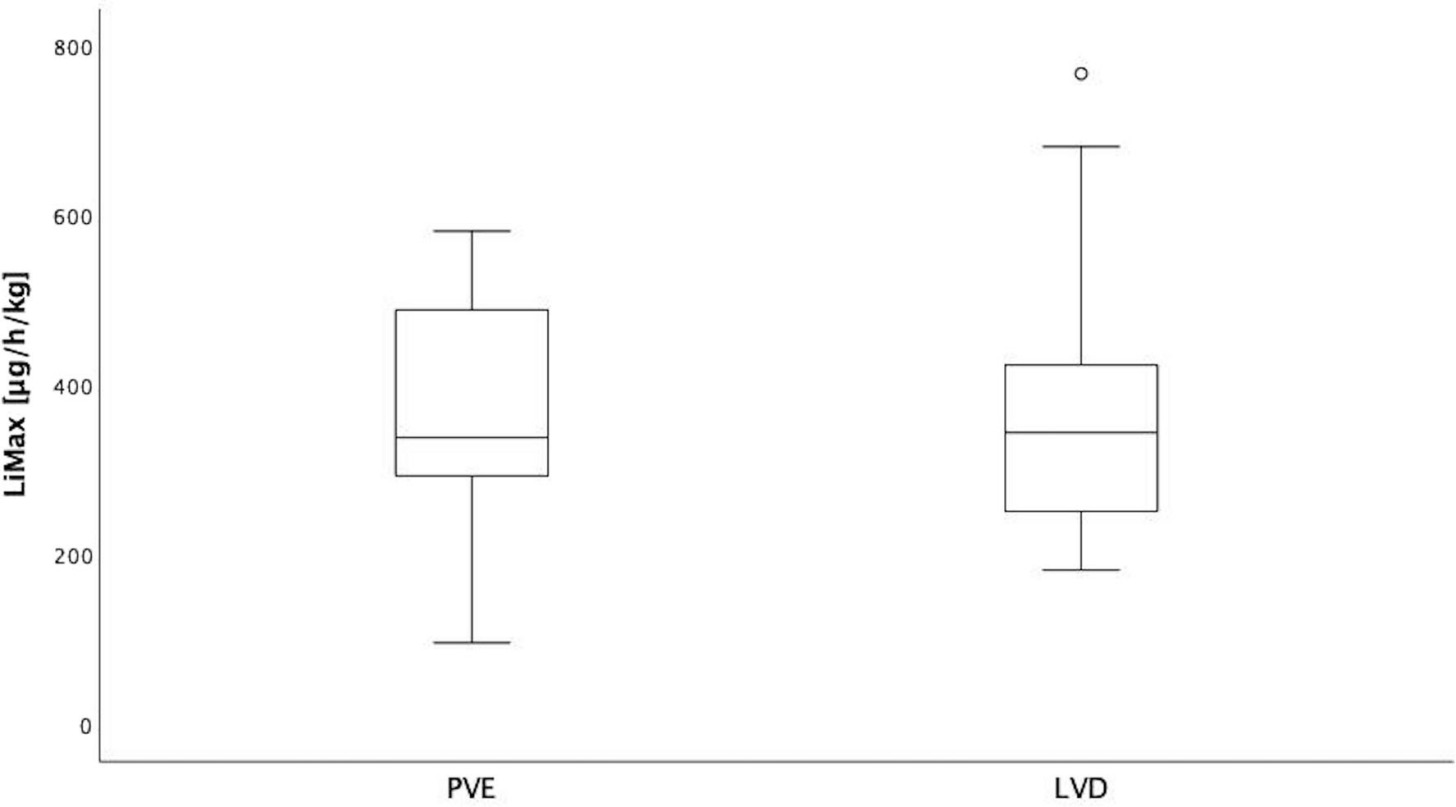
LiMAx scores before surgery. Liver function assessed by presurgical mean LiMAx scores shows no significant difference between the two groups (PVE: 363.4 ± 139.7 µg/h/kg, CI: 282.8–444.1 µg/h/kg; LVD: 377.1 ± 170.5 µg/h/kg, CI: 278.7–475.6 µg/h/kg; *p* = 0.820)

Presurgical mean LiMAx scores showed no significant difference in liver function between the two groups (PVE: 363.4 ± 139.7 µg/h/kg, CI: 282.8–444.1 µg/h/kg; LVD: 377.1 ± 170.5 µg/h/kg, CI: 278.7–475.6 µg/h/kg; *p* = 0.820) (Fig. 3).

### Technique of LVD

All embolizations (LVD and PVE alone) were completed as scheduled. In all 14 patients of the LVD group, a percutaneous transhepatic access to the right hepatic vein was used as cases with transjugular access were lost due to matching. There were no complications in the majority of interventions. Two complications occurred in the LVD group. In one patient, there was accidental embolization of the middle hepatic vein, and in another patient, a small amount of the liquid embolization material entered the vena cava via rapidly opening intrahepatic venous shunts during embolization. Both complications were classified as grade 1 according to CIRSE guideline [15].

In one case, an accessory right hepatic vein to segments V/VI was also embolized using a second plug and a mixture (2:1 ratio) of *n*-butyl-2-cyanoacrylate and ethiodized oil without any complication. In one case per group an additional plug was used for the PVE.

**Fig. 4:**
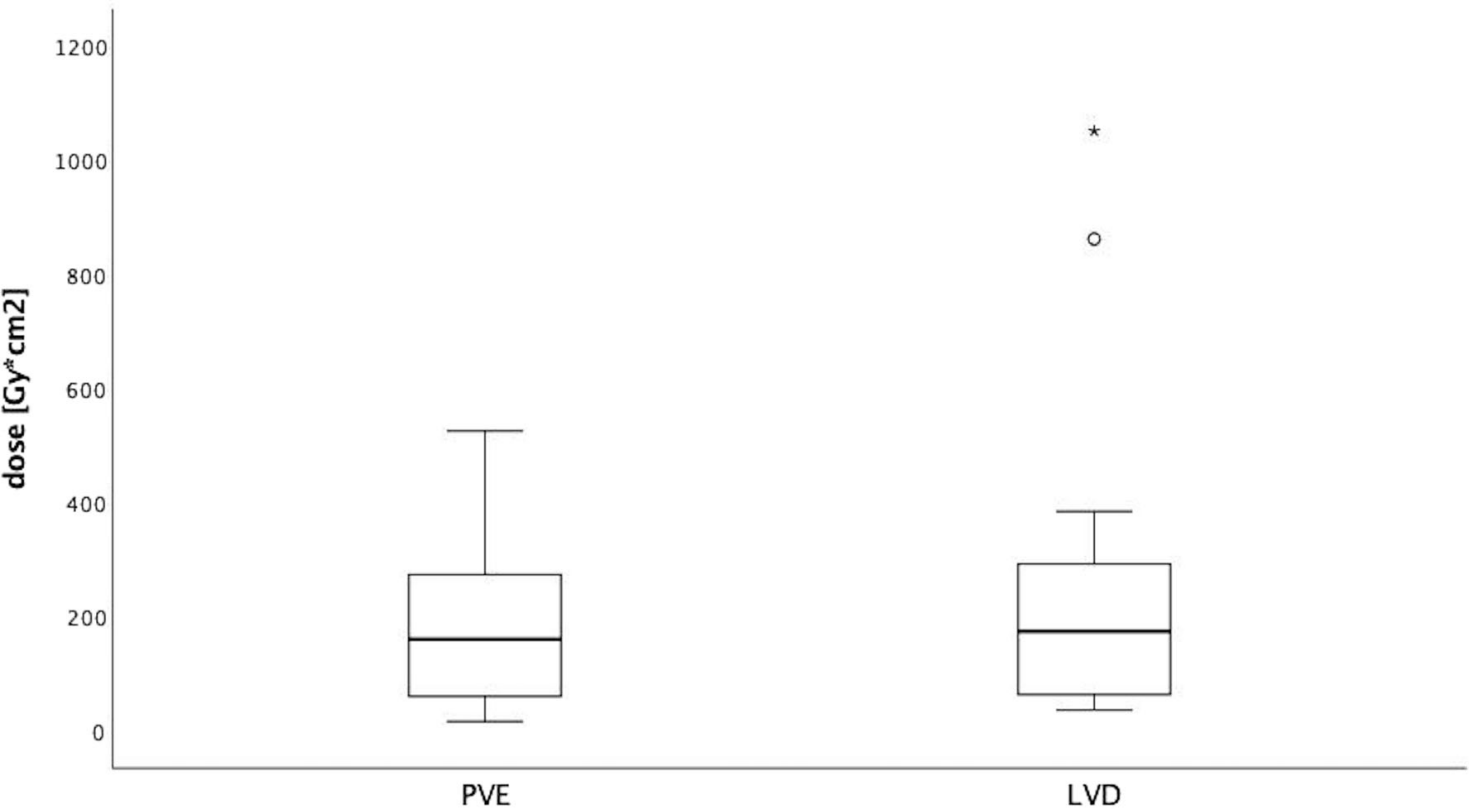
Radiation doses applied during interventions expressed as dose area product (Gray*cm^2^). Although a wider range of doses is apparent in the LVD group, mean applied doses do not differ significantly between the two groups (LVD: 268.9 ± 313.1 Gy*cm^2^, CI: 88.1–449.6 Gy*cm^2^; PVE: 186.1 ± 145 Gy*cm^2^, CI: 102.2–269.9 Gy*cm^2^; *p* = 0.431)

There were no significant differences in mean applied radiation doses between the two groups (LVD: 268.9 ± 313.1 Gy*cm^2^, CI: 88.1–449.6 Gy*cm^2^; PVE: 186.1 ± 145 Gy*cm^2^, CI: 102.2–269.9 Gy*cm^2^; *p* = 0.431) (Fig. 4). Nevertheless, because of the additional procedure, there is a trend toward higher doses for the LVD.

### Hypertrophy Rate

**Fig. 5:**
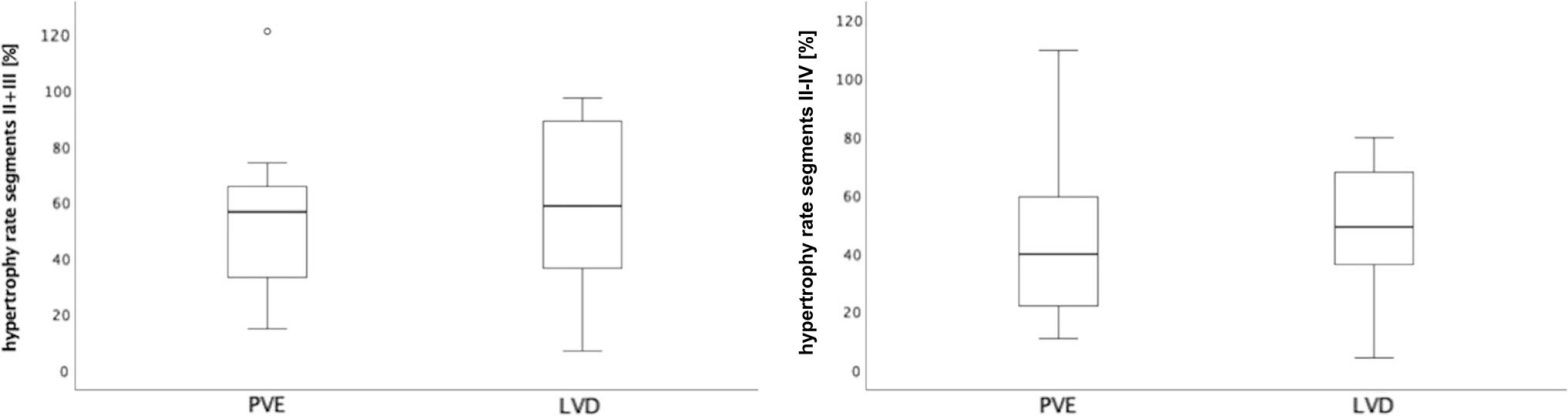
Hypertrophy rates of future liver remnant (FLR). LVD does not significantly improve mean relative hypertrophy of the future liver remnant (FLR). **a** Hypertrophy rate for segment II and III in % (PVE: 54.1 ± 27.6%, CI: 38.1–70%; LVD: 59 ± 29.6%, CI: 42–76.1%; p = 0.637); **b** hypertrophy rate for segment II, III and IV in % (PVE: 44.9 ± 28.9%, CI: 28.2–61.6%; LVD: 48.2 ± 22.2%, CI: 35.5–61; *p* = 0.719)

LVD did not lead to a significant improvement of the mean relative hypertrophy rate of the FLR in this study population (segments II + III: PVE: 54.1 ± 27.6%, CI: 38.1–70%; LVD: 59 ± 29.6%, CI: 42–76.1%; *p* = 0.637; segments II–IV: PVE: 44.9 ± 28.9%, CI: 28.2–61.6%; LVD: 48.2 ± 22.2%, CI: 35.5–61; *p* = 0.719) (Fig. 5).

## Discussion

Two complications occurred in the LVD group, including accidental embolization of the middle hepatic vein. Cases of erroneously embolized veins during sequential combined embolization have also been reported in the literature [16, 17]. The authors consider cone-beam CT (CBCT) to be the method of choice to ensure the best intraprozedural visualization of hepatic veins. Other investigators have suggested the use of a mobile CT unit [17, 18]. Of course, different views (lateral/oblique, etc.) and ultrasound can also be used for positioning.

LVD did not significantly improve the mean relative hypertrophy rate of the FLR in this study compared to controls. This finding disagrees with several studies reporting an advantage of combined embolization over PVE alone [6, 7, 19, 20], even when performed sequentially [16, 17]. Various factors may contribute to the discrepancies. Firstly, most published data were collected retrospectively, which may have led to a selection bias. In contrast, we used a prospective design for the LVD group. Secondly, part of the published studies investigated sequential embolization, while, in this study, combined embolization was performed in a single intervention. Thirdly, the method of embolization and other standards of surgical preparation sometimes differ considerably between centers. One group of investigators, for instance, not only used other materials for embolization but also consistently embolized the middle liver vein as well [6, 8]. Additionally, this center has a long and extended experience with PVE. The used standard technique is particle embolization followed by complete right portal vein blockage with coils or rarely plugs [4]. This technique has shown to have high FLR hypertrophy rates and a low rate of portal vein reperfusion which could at least partly be responsible for smaller differences between the groups in this study. Fourthly, only the volume increase of the FLR is amenable to direct comparison between studies, since, depending on the local standard procedure, different methods can be used to assess liver function [21–23]. However, the methods used to calculate hypertrophy of the FLR also differ among studies. In this study, manual measurement was used to calculate the relative volume increase of the FLR intraindividually.

In a retrospective study with a total of 50 cases (30 PVE vs. 20 LVD), a significant (*p* = 0.034) difference in median relative hypertrophy of the FLR was found (23% vs. 35%) at median hypertrophy intervals of 22 vs. 26 days [20]. However, the significant difference existed only in the subgroup (*n* = 19) with a hypertrophy period of up to 21 days [20]. This observation suggests that the potential benefit of LVD decreases with longer hypertrophy time, which would be consistent with the results of this study. To verify this observation, future studies could use sequential volumetric monitoring of hypertrophy, for example, by MRI.

This study has some limitations. Although this is a prospective study, only patients from a single center were included. In addition, a randomized study design was not approved by the local ethics committee, so only a comparison with a historical PVE population was possible. No sample size calculation in the sense of a statistical confirmatory approach was carried out for this exploratory analysis, resulting in a relatively small number of cases. Therefore, the study lacked sufficient statistical power to detect small effect sizes. Because combined LVD was not part of the standard procedure at this center before start of the study, a learning curve in the performance of the intervention must be assumed, which presumably influenced complication rates and applied radiation doses. Additionally, the patients in this study were not treated by one interventional radiologist only but by four very experienced interventional radiologists with more than 10 years of experience inter alia in PVE, which may better reflect clinical practice. In addition, sequential volumetry during the hypertrophy interval was not performed in this study. Therefore, the results do not allow a statement on the interim dynamics.

## Conclusions

LVD showed no significant advantages over the standard method (PVE alone) in terms of hypertrophy induction of the FLR before extended hepatectomy in this study population. These results differ from those of other studies, and further trials are needed to reach a consensus.

## Declarations

### Human and animal participants

This study been performed in accordance with the Declaration of Helsinki and was approved by the ethics committee of the Charité (EA2/073/18).
